# Community Composition of Predatory Hybotidae (Diptera: Empidoidea) in Winter Wheat Management Systems

**DOI:** 10.3390/insects16121263

**Published:** 2025-12-12

**Authors:** Julia Gitzel, Helge Kampen, Andreas Stark, Jörg Sellmann, Luca Marie Hoffmann, Jürgen Schwarz, Christian Ulrichs, Doreen Werner, Stefan Kühne

**Affiliations:** 1 Julius Kühn-Institut, Federal Research Centre for Cultivated Plants, 14532 Kleinmachnow, Germany; joerg.sellmann@julius-kuehn.de (J.S.); juergen.schwarz@julius-kuehn.de (J.S.); stefan.kuehne@julius-kuehn.de (S.K.); 2 Thaer-Institute, Humboldt-Universität zu Berlin, 14195 Berlin, Germany; christian.ulrichs@hu-berlin.de; 3 Friedrich-Loeffler-Institut, Federal Research Institute for Animal Health, 17493 Greifswald, Germany; helge.kampen@fli.de; 4 Zentralmagazin Naturwissenschaftlicher Sammlungen, Martin-Luther-Universität Halle-Wittenberg, 06108 Halle, Germany; andreas.stark@zns.uni-halle.de; 5 Senckenberg German Entomological Institution, 15374 Müncheberg, Germany; 6 Leibniz Centre for Agricultural Landscape Research, 15374 Müncheberg, Germany; doreen.werner@zalf.de

**Keywords:** agrobiodiversity, biological pest control, Diptera, Hybotidae, pesticide avoidance, predator–prey relationships

## Abstract

Fast-running flies (Diptera: Hybotidae) play an important role as predators in agricultural landscapes. This semi-field study examined the effects of pesticides on Hybotidae communities and their role in natural pest control in three winter wheat management systems (organic, conventional, and hybrid (no chemical synthetic pesticides with optimized use of nitrogen fertilizers)) in Brandenburg, Germany. To evaluate the impact of management practices, sweep netting and eclector trapping were carried out over three years (2020–2022) at the plot scale. Hybotidae abundance fluctuated across the management systems and collection methods, with no consistent trend linked to pesticide use. However, an increase in the abundance of Hybotidae in 2022, especially in eclector trapping, indicated that year-to-year fluctuations were pronounced and likely driven by environmental factors, such as climate and soil moisture, rather than management practices. The community structure showed a high degree of similarity among all management systems, but species diversity displayed pronounced interannual variation, suggesting complex ecological interactions. Sweep netting collections indicated positive predator–prey associations in every management system, pointing to a generally stable trophic structure. The study also demonstrates that using multiple insect collection methods is crucial for accurately assessing insect diversity and abundance. Further research is needed to fully understand species diversity, predator–prey dynamics, and their implications for sustainable agriculture.

## Simple Summary

This study examined the community of Hybotidae, a brachyceran family of predatory flies, and their role in natural pest control within different winter wheat management systems (organic, conventional, and hybrid (no chemical synthetic pesticides with optimized use of nitrogen fertilizers)). The semi-field study aimed to explore how agricultural practices, particularly pesticide avoidance, affect Hybotidae communities, thereby contributing to the broader discourse on sustainable agriculture and the conservation of agricultural biodiversity. Results showed fluctuating Hybotidae population densities across different management systems and years, with no consistent link to pesticide application. The complex interactions may be strongly influenced by other factors, such as environmental ones like climate, soil moisture, and prey availability. The results advocate for long-term studies to better understand ecological dynamics and argue for agricultural strategies that balance productivity with environmental preservation.

## 1. Introduction

Dipteran insects are a crucial component of all ecosystems. They exhibit a remarkable diversity, including a vast array of species that occupy a wide range of ecological niches [1]. In the context of agricultural ecosystems, Diptera play a multifaceted role. Some dipteran species are pests, marring plant health and reducing agricultural yield [2,3,4]. Other families contribute to pollination, decomposition, and biological control of pests [5,6,7,8,9,10]. At the same time, Diptera are important indicators of agrobiodiversity, as their presence and diversity can reflect the overall health and balance of the agricultural ecosystem [11].

A significant element within the order Diptera is the Hybotidae (“fast running flies”). They are globally distributed and exhibit extensive diversification regarding morphology and coloration [12,13]. Species of numerous genera are visitors of flowering weeds in agricultural fields during their adult stage, presumably to obtain nectar, while flower visits by other tribes, such as *Platypalpus* and *Drapetis*, are considered accidental, as these species are primarily predatory [14].

Hybotidae participate in biological pest control, as they are predators of a wide variety of soft-bodied arthropods [14,15,16]. The predatory flies, especially *Platypalpus* and *Tachydromia*, are natural enemies of plant pests in wheat, e.g., Cecidomyiidae, Agromyzidae, and Sciaridae [17,18,19]. For the particularly small genus *Stilpon*, larval stages of thrips and leafhoppers were detected as prey [19]. Overall, adult Tachydromiinae are natural enemies of many agricultural pests and hold promise as effective biocontrol agents [20,21]. The small, fast-running flies, which are only 1–5 mm long, are predatory both as larvae and adults and often occur in high abundances in all cereal ecosystems [20,22]. The larval stages remain poorly understood and are known to inhabit diverse ecological niches, including aquatic, semiterrestrial, and terrestrial environments such as moist soil and decomposing plant material. They exhibit predatory behavior, feeding on a variety of small invertebrates [15,20,21]. The predation activities of the Hybotidae contribute to minimizing pest damage to the wheat crop, offering the possibility of reducing the reliance on chemical pesticides for pest management. This natural pest control mechanism may not only support wheat production, but it also aligns with sustainable farming practices and environmental conservation efforts. The diverse contributions of Hybotidae in wheat production emphasize the need for conservation and preservation of their habitats within agricultural landscapes.

In recent years, studies on the effects of pesticides on insect occurrence, biodiversity, and behavior have focused predominantly on bees [23,24,25]. Only a few investigations have considered the impact of insecticides on pollinating hoverflies (Syrphidae) and tachinid flies (Tachinidae) [26,27,28]. By contrast, other Diptera families are largely neglected in current environmental risk assessments.

To assess the presence and composition of Diptera groups in a given habitat, the application of suitable sampling and identification methods is essential. The effectiveness of each approach depends on the target taxa, as different groups respond to distinct collection techniques [29,30].

Sweep netting, which involves passing a net through vegetation to capture active arthropods, is widely used for collecting species inhabiting upper vegetation layers. It efficiently samples a broad spectrum of taxa, particularly diurnal and mobile species occurring in open habitats [31]. Hybotidae are typically well represented in sweep netting samples, as many species perch on vegetation and display sit-and-wait predatory behavior [32].

Conversely, ground-dwelling or cursorial families such as Tachydromiinae may be underrepresented by this method. For such taxa, eclector traps offer a complementary approach, providing density estimates based on individuals emerging from a defined substrate area rather than potential migrants [33]. Several studies have shown that eclector trapping is an effective tool for assessing Hybotidae assemblages living and hunting near the ground [34,35,36]. They additionally give a reliable estimate of the soil-dwelling larvae.

Given the impact of biodiversity on the global economy and the importance of ecosystem services for human well-being, adopting nature-based farming practices that sustain or enhance productivity while promoting landscape, biodiversity, and soil conservation is essential. Such approaches are crucial for ensuring the long-term sustainability of agricultural systems, aligning with the goals of the EU Biodiversity Strategy for 2030, and enhancing their resilience to climate change [37].

Research into novel agricultural practices, which avoid chemical synthetic pesticides but optimize nitrogen fertilization for stable yields, is therefore essential. To address these challenges, a new hybrid cropping system (NOcsPS) was developed, combining key principles of organic farming and integrated pest management (IPM) [38]. The NOcsPS management system avoids all chemical synthetic pesticides, including insecticides, herbicides, and fungicides. It combines established and innovative technologies with agroecological practices to strengthen natural regulatory processes. At the same time, it incorporates optimized mineral fertilization and non-chemical crop protection strategies to ensure stable and sustainable yields. The present study evaluated its impact on biodiversity conservation and ecosystem balance, with a focus on Hybotidae as a biodiversity indicator in the new hybrid management system.

Hybotidae may be sensitive to chemical synthetic pesticides, although this has not been empirically demonstrated.

Based on three winter wheat management systems, this semi-field study aimed to determine the effects of avoidance of chemical synthetic pesticides with simultaneous optimized nitrogen application on (1) the abundance of Hybotidae in the different management systems; (2) species diversity in wheat management systems; and (3) predator–prey relationships between Hybotidae as predators and Agromyzidae, Chloropidae, Cecidomyiidae, and Sciaridae as their prey.

## 2. Materials and Methods

### 2.1. Study Area and Agricultural Management

Insect collections were carried out at the experimental field site of the Julius Kühn-Institute in Dahnsdorf (federal state of Brandenburg, Germany: N52.108494, E12.636338), which covers an area of 38 ha. The site’s elevation ranges from 77 to 85 m above sea level. The loamy soil exhibited a mean quality of 48 points [39] and comprised 57.9% sand, 37.5% silt, 4.6% clay, and 1.4% organic matter, with a pH of 5.8. The location experienced an average annual temperature of 9.7 °C and an average annual precipitation of 564 mm over the period from 1997 to 2024.

We investigated the effect of avoiding chemical synthetic pesticide application on Hybotidae communities over three vegetation seasons (early May to late June in 2019/2020, 2020/2021, and 2021/2022). The study compared three management systems that differed in management intensity [40]: organic, conventional, and a hybrid system, ‘NOcsPS’ (No chemical synthetic Pesticides).

The study utilized a six-year crop rotation design consisting of winter wheat I (variety: Achim), silage maize (variety: Ronaldinio), winter rye (variety: KWS Binntto), pea (variety: Astronaute), winter wheat II (variety: RGT Reform), and spring barley (variety: Leandra). In the organic management system, spring barley was replaced by clover grass (variety: Semopur 2.2). Each crop was cultivated in four replicates, with each replicate containing three plots of the respective crop within the study field. Each plot measured 5 m × 10 m, totaling 50 m^2^. The plots within each replicate were spaced 10 m apart. Each cropping system received treatment regimens adapted annually in terms of fertilizer application and pesticide use. Our investigation focused exclusively on winter wheat (variety: RGT Reform). In 2019/2020, the organic management system was sown with the variety Govelino, which was changed to RGT Reform in the subsequent years to eliminate the influence of varietal differences.

**Figure 1:**
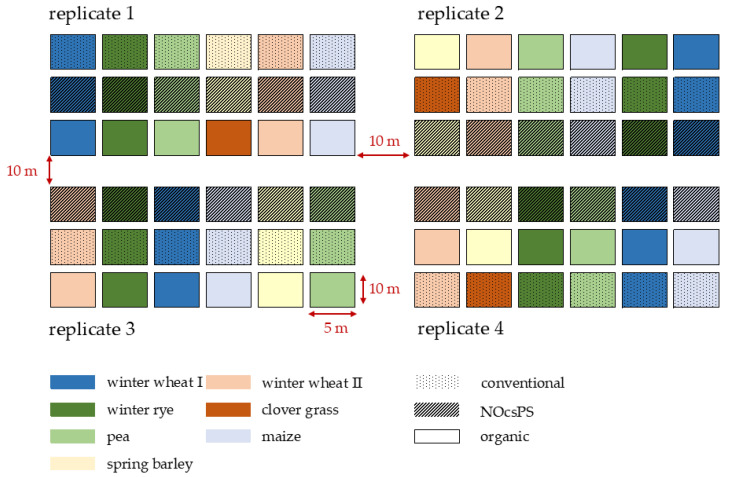
Experimental design of the six-year crop rotation study in harvest year 2020/2021 for organic, NocsPS, and conventional management systems. Dotted areas in the figure represent conventional plots, striped areas represent NOcsPS plots, and blank areas represent organic plots.

In summary, 12 plots were sampled (four per management system) every year. The study employed a randomized block design to minimize edge effects and variations in soil fertility and water-holding capacity, resulting in different spatial positions of the plots across the replicates. The position of each crop within the replicates was rotated annually (Figure 1).

Across all three study years, plots were uniformly managed using mechanized cultivation, sowing, and weed control, conducted between 30th September and 4th October. The agricultural process of the harvest year began with general plot preparation, followed by primary soil tillage using plowing. Seeds were precisely sown using a rotary harrow combined with a seed drill. All operations, including soil cultivation, sowing, and mechanical weed control, were conducted within the designated plots (Table S1).

Sowing rates of wheat varied: 360 grains/m^2^ for the conventional management system, 380 grains/m^2^ for the organic management system (to mitigate potential losses from mechanical weed control), and 250 grains/m^2^ for the NOcsPS management system. In the conventional and organic management system, the seed drill (Amazone AD (drill combination Amazone special AD/KE 2500)), with a working width of 2.5 m, sowed two strips per plot, resulting in a total width of 5 m. Drilling was conducted with row spacings of 12.5 cm. The sowing pattern consisted of three seed rows, followed by two empty rows (serving as lanes), then ten seed rows, two lanes, six seed rows, two lanes, ten seed rows, two lanes, and finally three seed rows (Table S2). Regarding seed distribution, the collaborative research project compared normal sowing with approximate equidistant sowing (single-grain placement), which differs in row spacing and seed rate configuration. Potential effects of sowing density were addressed in a separate study. Due to single-grain placement, the NOcsPS management system had a different sowing pattern, with row widths of 15 cm, and a seed drill working width of 3 m. The extra 1 m width of the plot was milled. The drilling scheme consisted of two seed rows, followed by two lanes, eight seed rows, two lanes, five seed rows, two lanes, eight seed rows, two lanes, and finally two seed rows (Table S2).

The seed grains of the conventional management system were pickled with Landor CT in 2019/2020 (composition: fludioxonil 25 g/L (2.4% by weight), difenoconazole 20 g/L (1.9 wt.%), tebuconazol 5 g/L (0.5 wt.%)) and Rubin TT (composition: prochloraz 38.6 g/L (42 g/L copper chloride complex), pyrimethanil 42 g/L, triticonazole 25 g/L) in 2020/2021 and 2021/2022.

The application of fertilizers and pesticides differed among management systems and also varied between experimental years. Fertilizers and pesticides were applied from the outside of the plot to avoid driving through the crop. Fertilization was carried out annually in March, applying 40 kg/ha of Kieserit (containing 25% MgO, equivalent to 15.1% water-soluble Mg, and 52% SO_3_, equivalent to 20.8% water-soluble S) in 2020 and 100 kg/ha in 2021 and 2022, including 8 kg/ha of sulfur (S) and 10 kg/ha of magnesium oxide (MgO) in 2020 as opposed to 20 kg/ha S and 25 kg/ha MgO in 2021 and 2022. Nitrogen fertilization in the conventional management system took place annually in March using KAS 27% N (calcium ammonium salts with 13.5% nitrate nitrogen and 13.5% ammonium nitrogen) and pure nitrogen (370 kg/ha KAS with 100 kg/ha N in 2020 and 2021, 296 kg/ha KAS with 79.9 kg/ha N in 2022). Additional nitrogen was added at the end of April (148 kg/ha KAS, including 40 kg N/ha in 2020 and 2021, 111 kg/ha KAS consisting of 30 kg N/ha in 2022).

The conventional management system was managed in accordance with IPM principles. Therefore, it involved applying pesticides only after weed or pest thresholds were exceeded, following weekly pest assessments as recommended by regional extension services. This resulted in the application of insecticides for the harvest year 2021/2022 as follows: in November 2021 (wheat growth stage (GS) 12/13), the larvae of the ground beetle (*Zabrus tenebrioides* (Goeze, 1777)) were controlled using the insecticide Karate Zeon (75 mL/ha; 100 g/L active ingredient lambda-cyhalothrin) in replicates 1 and 4, but not in replicates 2 and 3, where pest thresholds had not been exceeded. The same treatment was administered in March 2022 (GS 23) for replicate 2, after pest thresholds were surpassed. Herbicides were applied post-emergence, following wheat germination. The herbicide Bacara Forte (1 L/ha; active ingredients 120 g/L diflufenican, 120 g/L flufenacet, 120 g/L flurtamone) was applied at GS 13 in harvest year 2019/2020. In harvest year 2020/2021, the herbicide Trinity was utilized once at GS 12 at 1.5 L/ha (active ingredients 300 g/L pendimethalin, 250 g/L chlortoluron, 40 g/L diflufenican) and again in 2021/2022 at GS 12 at an application rate of 2.0 L/ha. Fungicidal treatment against *Septoria tritici* was necessary in harvest years 2019/2020 and 2020/2021. In 2020, 1.25 L/ha of Input Classic fungicide (active ingredients 160 g/L prothioconazole, 300 g/L spiroxamine) was applied at GS 37. In 2021, a combination of Revytrex (1.2 L/ha; active ingredients 66.7 g/L fluxapyroxad, 66.7 g/L mefentrifluconazole) and Comet (0.4 L/ha; active ingredient 200 g/L pyraclostrobin) was applied at GS 43.

The organic management system adhered to Regulation (EU) 2018/848 and its corresponding implementing regulations [41], which aim to prohibit the use of chemical synthetic pesticides. Nitrogen fertilization was achieved through the cultivation of green manures. To enhance soil nitrogen levels, peas were used as the preceding crop and clover grass as the succeeding crop in the rotation design. Mechanical weed control and management measures are detailed in Table S1. Further fertilization was applied annually in March using 40 kg/ha Kieserit, containing 8 kg/ha S and 10 kg/ha MgO in the harvest year 2019/2020 and 100 kg/ha Kieserit comprising 20 kg/ha S and 25 kg/ha MgO in 2020/2021 and 2021/2022. Weekly monitoring and assessments for pests were carried out, but no treatment was applied due to the lack of organic pesticides effective against ground beetles, aphids, and fungi. Chemical synthetic insecticides and fungicides were not used. Mechanical weed control was carried out once in autumn prior to germination (GS 21/22) and twice in spring (GS 24/25) as a replacement for herbicide application, with the exception of the harvest year 2019/2020, during which no blind weeding was performed in the autumn of the preceding year.

In the NOcsPS management system, nitrogen fertilization was reduced by 30% compared to the conventional management system, under the assumption of a corresponding yield decrease [42]. Because the NOcsPS system is not yet widely implemented, standard guidelines for fertilization were lacking. Consequently, fertilization rates were set according to long-term experience from the experimental field in Dahnsdorf. Fertilization was performed using 40 kg/ha Kieserit, with 8 kg/ha S and 10 kg/ha MgO (in 2019/2020). In harvest years 2020/2021 and 2021/2022, the fertilization application was increased to 100 kg/ha Kieserit, including 20 kg/ha S and 25 kg/ha MgO. Nitrogen fertilization was applied as follows: in 2020, 260 kg/ha of calcium ammonium nitrate (KAS) providing 70 kg/ha N took place in March, followed by 104 kg/ha KAS with 28 kg/ha N in April. In 2021, 185 kg/ha KAS containing 50 kg/ha N was applied in early March, and an additional 88 kg/ha KAS with 23.8 kg/ha N was applied in late March and late April. In 2022, the application of 207 kg/ha KAS with 55.9 kg/ha N took place in GS 25, followed by 44 kg/ha KAS consisting of 11.9 kg/ha N in GS 31. Chemical synthetic insecticides, fungicides, and herbicides were not used. Mechanical weed control methods were adapted to those employed in the organic management system to eliminate the need for herbicides (Table S1).

### 2.2. Entomological Investigations

From early May to late June of each study year, sweep netting and eclector trapping were used to study the Hybotidae assemblage. Sampling activities were conducted each week consistently on the same day, with the order of sampled plots randomized weekly.

#### 2.2.1. Sweep Netting

Sweep netting was conducted annually on eight occasions on dry days (from 10 a.m. to 1 p.m.) or during dry periods to coincide with peak insect flight activity [43,44]. The sweeping net, characterized by a diameter of 0.3 m and a 0.65 m handle, was systematically employed over designated lanes (organic and conventional: row numbers 16/17 and 24/25 (distance to the border of the plot: ca. 1.9 m); NOcsPS: row numbers 13/14 and 21/22 (distance to the border of the plot: ca. 2 m)) (Table S2). Each catch involved 25 double scoops along these lanes, directly above the wheat stand, stripping the vegetation. The net was made of white fine gauze (105 μm mesh size), reinforced with a leather frame, and had a depth of 50 cm. During the three-year investigation, a total of 204 sweep netting samples were collected. The collected insects were subsequently recovered from the net using an aspirator and transferred into 50 mL PE bottles containing 70% ethanol.

#### 2.2.2. Eclector Trapping

The eclector consists of a pyramid-shaped collecting chamber (tent) mounted above a collection container filled with a preservative solution (1:1 mixture of tap water and ethylene glycol (70 mL per trap), with a drop of detergent added to reduce the surface tension. The tent, constructed from 1 × 1 mm mesh, intercepts insects moving upward from the soil or along the ground and directs them into the collection container. The trap is placed directly on the soil surface, with the base slightly embedded to ensure efficient capture. The eclectors, with a footprint of 0.25 m^2^, were positioned 2 m from the border inside the plots at the start of May and shifted approximately 1 m into the plot every 14 days until the end of June. During this process, the wheat stalks were shortened by approximately 10–15 cm upon reaching the height of the eclector trap. The stalks were positioned beneath the trap to only minimally alter the conditions for the Hybotidae and the other Diptera considered. All samples obtained with this collection method were transferred into 50 mL PE bottles and preserved in 70% ethanol.

### 2.3. Morphological Insect Identification

To calculate the Hybotidae abundance and to examine their predator–prey relationships, the collected insects were categorized into Hybotidae and prey for each management system and collection method, counted, and organized chronologically by collecting date. The following dipteran families were considered as prey: Agromyzidae, Chloropidae, Cecidomyiidae, Sciaridae, and all other encountered Nematocera with a body size of up to 7 mm.

All Hybotidae collected via sweep netting and eclector trapping were morphologically identified to the species level utilizing established taxonomic keys [21,45,46]. Given the impracticality of conducting a taxonomic assessment at the species level with the high number of Agromyzidae, Chloropidae, Cecidomyiidae, and Sciaridae collected, the morphological determination of those was made to family or suborder level only [47,48].

### 2.4. Genetic Insect Identification

Collected Hybotidae that could not be reliably determined morphologically were identified genetically by COI (cytochrome c oxidase subunit 1 gene) barcoding [49], using primers LCO1490 (forward: 5′-GGTCAACAAATCATAAAGATATTGG-3′) and HCO2198 (reverse: 5′-TAAACTTCAGGGTGACCAAAAAATCA-3′) [50]. DNA was extracted from single legs or equivalent pieces of tissue. The 710 bp DNA fragment was sequenced unidirectionally with one of the PCR primers by a sequencing service (Eurofins, Konstanz, Germany). Obtained DNA sequences were submitted to BOLD (https://id.boldsystems.org) and/or GenBank (https://blast.ncbi.nlm.nih.gov/Blast.cgi?PAGE_TYPE=BlastSearch; accessed on 31 January 2024) identification engines. Sequence identities of ≥99% were accepted as representing the correct species. Barcoding was performed on six taxonomically ambiguous individuals: three were identified to species level, while the remaining three individuals were recorded as Hybotidae sp. and included only in abundance counts, not in diversity indices.

### 2.5. Calculation of Relative Hybotidae Abundance

After thoroughly counting the collected total number of Hybotidae for each plot, mean and median values for each collection date were calculated within the management systems according to the collection method. This calculation involved dividing the total number of individuals across all plots by the number of collections.

### 2.6. Alpha Diversity Indices

The species diversity of Hybotidae was assessed using the Shannon index (Hs) and evenness (E) [51]. The Shannon index is a mathematical measure utilized to quantify the diversity within an ecosystem, considering both the number of species and the relative abundance of each species within the dataset.

The Shannon index is calculated as $Hs=-\sum_{i=1}^{s}pi*ln\;pi$, with pi representing the proportion of species (*i*) relative to the total number of species, indicating the relative frequency of each species [52].

Evenness is a parameter to compare different communities, quantifying the individual distribution within a population. The calculation of evenness is based on the following formula: $E=\frac{Hs}{\ln S}$, with Hs equaling the diversity related to the number of species, and *S* representing the total number of species [51].

The Berger–Parker index (d) is commonly employed in ecology for evaluating species dominance within a community [53]. This index specifically measures the dominance of the most abundant species. The Berger–Parker index is calculated using the formula d=Nmax/N, with Nmax representing the abundance of the most prevalent species, and *N* representing the total number of individuals in the sample.

### 2.7. Measurement of Soil Moisture

The soil moisture was measured using the soil sensor device of the weather station (Umwelt-Geräte-Technik GmbH Müncheberg, Müncheberg, Germany) at the experimental field site of the Julius Kühn-Institute in Dahnsdorf. Three sensors were used, which recorded the moisture every 10 min in 10 cm, 20 cm, 30 cm, and 60 cm soil depth.

### 2.8. Statistics

Data analysis was performed using the statistical software SAS 9.4 (SAS, Cary, NC, USA).

Generalized linear mixed models (GLMMs) were fitted using the PROC GLIMMIX procedure to evaluate the effects of management systems on Hybotidae abundance and alpha diversity indices (Shannon index, evenness, and Berger–Parker index).

For each model, ‘management system’ (bew_art) was included as a fixed effect. The potential interaction between ‘year’ and ‘management system’ was initially tested but did not contribute significantly to the model and was therefore not retained as a fixed effect. Instead, ‘collection date’ (fang_datum), ‘year’ (jahr), ‘replication’ (wdh), and their interaction (jahr × wdh) were treated as random effects to account for temporal variation and repeated sampling across years within the hierarchical experimental design. A negative binomial distribution (dist = nb) was specified to model the count data and to account for non-normality in Shannon diversity values. A log-normal distribution (dist = logn) was assumed for evenness data, as values were right-skewed and continuous. Denominator degrees of freedom were estimated using the Kenward-Roger method (ddfm = kr).

Least-squares means (LS-means) were computed for all fixed effects. Pairwise comparisons among management systems were adjusted using the SIMULATE method (adjust = simulate, seed = 12,345), which maintains the overall significance level at α = 0.05. This procedure is suitable for unbalanced data and heterogeneous variances and is less conservative than the TUKEY adjustment [54]. For the Berger–Parker index, a Tukey adjustment was applied to pairwise comparisons.

To assess temporal variation in Hybotidae abundance independently of the management systems, a generalized linear mixed model (GLMM) was fitted using the PROC GLIMMIX, including ‘year’ (jahr) as a fixed effect, while ‘collection date’ (fang_datum) and ‘management system’ (bew_art) were treated as random effects. Again, a negative binomial distribution (dist = nb) with a log link was used, and denominator degrees of freedom were estimated using the Kenward-Roger method (ddf = kr). Least-squares means (LS-means) were calculated for the factor ‘year’, and pairwise comparisons were adjusted using the SIMULATE method (adjust = simulate, seed = 12,345), maintaining an overall significance level of α = 0.05.

To explore relationships between Hybotidae as predators (ANZAHL_INDIV_HYBOTIDAE) and Agromyzidae, Chloropidae, Cecidomyiidae, Sciaridae, and other Nematocera (<7 mm body size) as prey (beute), a Spearman’s rank correlation with Fisher’s z-transformation to calculate 95% confidence intervals and *p*-values was conducted [55].

## 3. Results

### 3.1. Hybotidae Abundance

#### 3.1.1. Relative Hybotidae Abundance in Sweep Netting Across Management Systems

**Figure 2:**
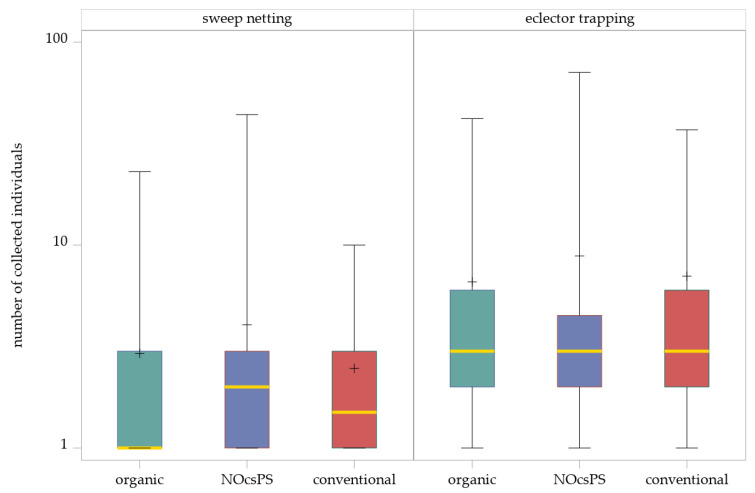
Effects of management systems on Hybotidae abundance as determined by sweep netting and eclector trapping. Results were generalized with linear models (GLMMs) using ‘management system’ as a fixed effect and ‘year’, ‘collection date’, and ‘replicate’ as random effects on the abundance of Hybotidae. The yellow middle line represents median values. The upper and lower lines represent the first and third quartiles. The lower and upper hinges represent maximum and minimum values. ‘+’ represents the mean value. For better visualization, the *y*-axis was scaled logarithmically.

The generalized linear mixed model (GLMM) with a negative binomial distribution indicated no significant effects of the management system on the abundance of Hybotidae in sweep netting (F_2,128_ = 1.73, *p* = 0.1808). The SIMULATE method for multiple testing (α = 0.05) showed that none of the comparisons of three management systems were significant (Table 1; Figure 2).

Although management system effects were not statistically significant, the NOcsPS management system showed the highest number of individuals (total: 178; mean ± SD: 4.05 ± 7.23). In the organic management system, a total of 155 individuals were collected (mean ± SD: 2.92 ± 4.05), whereas the conventional management system showed comparatively lower numbers, with only 84 individuals collected across the three experimental years (mean ± SD: 2.47 ± 2.39).

#### 3.1.2. Hybotidae Abundance in Eclector Trapping Across Management Systems

For the eclector trapping data, the GLMM likewise revealed no significant effect of the management system (F_2,73.01_ = 0.44, *p* = 0.6460). The SIMULATE method for multiple comparisons confirmed that no significant differences occurred (Table 1; Figure 2). The highest number of individuals was determined in the NOcsPS management system (total: 283; mean ± SD: 8.84 ± 16.21). In contrast, more individuals were collected in the conventional management system (total: 260; mean ± SD: 7.03 ± 9.07) than in the organic management system (total: 224; mean ± SD: 6.59 ± 9.73).

#### 3.1.3. Hybotidae Abundance Across Years

Although no significant management system effects were found, the total number of Hybotidae collected by eclector trapping varied strongly between years (Table 2). In season of 2022, markedly higher abundances (number of individuals: 605; mean ± SD: 13.75 ± 16.06) were recorded in all management systems compared with 2020 (number of individuals: 38; mean ± SD: 1.81 ± 1.7) and 2021 (number of individuals: 24; mean ± SD: 3.26 ± 1.87).

This effect was less pronounced in the sweep netting collections. Fewer individuals were collected in 2020 (total = 50; mean ± SD: 1.52 ± 0.87) compared with the following years, while the number of individuals in 2021 (total = 194; mean ± SD: 3.66 ± 6.33) and 2022 (total = 173; mean ± SD: 3.84 ± 5.03) differed only slightly.

**Figure 3:**
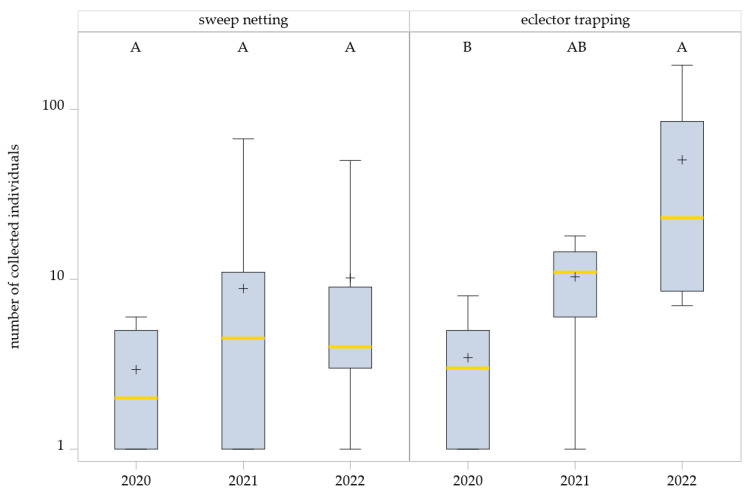
Effects of years on Hybotidae abundance as determined by sweep netting and eclector trapping. Results were generalized with linear models (GLMMs) using ‘year’ as a fixed effect and ‘collection date’ and ‘management system’ as random effects on the abundance of Hybotidae. The yellow middle line represents median values. The upper and lower lines represent the first and third quartiles. The lower and upper hinges represent maximum and minimum values. ‘+’ represents the mean value. Boxes with the same letter within the year are not significantly different. For better visualization, the *y*-axis was scaled logarithmically.

The generalized linear mixed model (GLMM) revealed no significant effect of the year on Hybotidae abundance in sweep netting (F_2,21.72_ = 1.77, *p* = 0.1949). Although sweep netting captures showed a slight upward trend across years, variability within years was high, and differences were not statistically significant: 2020 vs. 2021, *p* = 0.3243; 2020 vs. 2022, *p* = 0.2130; and 2021 vs. 2022, *p* = 0.9464 (Figure 3).

In contrast, the GLMM for eclector trapping data detected a significant year effect on Hybotidae abundance (F_2,9.31_ = 5.95, *p* = 0.0216). Pairwise comparisons (SIMULATE-adjusted) indicated significantly higher abundances in 2022 than 2020 (*p* = 0.0183), whereas differences between 2021 and 2022 were not significant (*p* = 0.2885). Additionally, comparisons between 2020 and 2021 also showed no significant differences (*p* = 0.934) (Figure 3).

### 3.2. Alpha Diversity Measures

#### 3.2.1. Shannon Index, Evenness, and Berger–Parker Index in Sweep Netting

**Figure 4:**
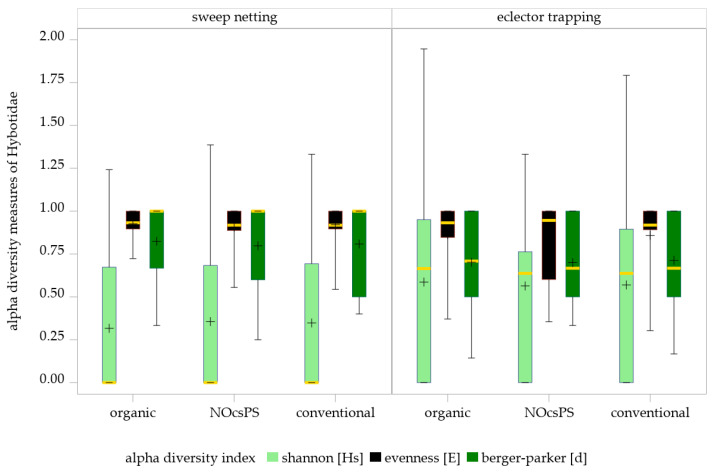
Effects of management systems on alpha diversity measures as determined by sweep netting and eclector trapping. Results were generalized with linear models (GLMMs) using ‘management system’ as a fixed effect and ‘year’, ‘collection date’, and ‘replicate’ as random effects on the diversity indices of Hybotidae. The yellow middle line represents median values. The upper and lower lines represent the first and third quartiles. The lower and upper hinges represent maximum and minimum values. ‘+’ represents the mean value. For better visualization, the *y*-axis was scaled logarithmically.

No significant management system effects were detected on Shannon diversity (GLMM results: F = 0.04; df = 2,28; *p* = 0.9651), evenness (GLMM results: F = 0.45; df = 2,51.43; *p* = 0.6396) or Berger–Parker index (GLMM results: F = 0.14; df = 2,17.4; *p* = 0.8666) in sweep netting (Table 3; Figure 4). Applying the SIMULATE method for multiple testing (α = 0.05), none of the pairwise comparisons among the three management systems reached statistical significance. The NOcsPS management system achieved the highest values on Shannon diversity (Hs ± SD: 0.36 ± 0.41), but exhibited the lowest values on evenness and Berger–Parker index (E ± SD: 0.91 ± 0.13; d ± SD: 0.80 ± 0.24). Although the organic management system performed with the lowest Shannon index (Hs ± SD: 0.32 ± 0.40), it showed the highest values for the other two indices (E ± SD: 0.93 ± 0.08; d ± SD: 0.82 ± 0.22). The conventional management system consistently occupied an intermediate position across all indices (Hs ± SD: 0.35 ± 0.43; E ± SD: 0.92 ± 0.12 d ± SD: 0.81 ± 0.23).

#### 3.2.2. Shannon Index, Evenness, and Berger–Parker Index in Eclector Trapping

The GLMM results of eclector trapping data indicated no significant effects of the management system on Shannon index (F = 0.02; df = 2100; *p* = 0.9780), evenness (F = 0.09; df = 2,54.46; *p* = 0.9123), and Berger–Parker index (F = 0.2; df = 2,85.45; *p* = 0.8219) of Hybotidae. The SIMULATE method for multiple testing (α = 0.05) showed that none of the comparisons of three management systems were significant (Table 3; Figure 4).

These findings are consistent with the diversity indices of the management systems, which revealed only slight variations among them: NOcsPS (Hs ± SD: 0.56 ± 0.41; E ± SD: 0.84 ± 0.23; d ± SD: 0.70 ± 0.25), organic (Hs ± SD: 0.59 ± 0.53; E ± SD: 0.86 ± 0.19 d ± SD: 0.70 ± 0.28), and conventional (Hs ± SD: 0.57 ± 0.51; E ± SD: 0.86 ± 0.21; d ± SD: 0.71 ± 0.27).

#### 3.2.3. Community Structure of Hybotidae Species

**Figure 5:**
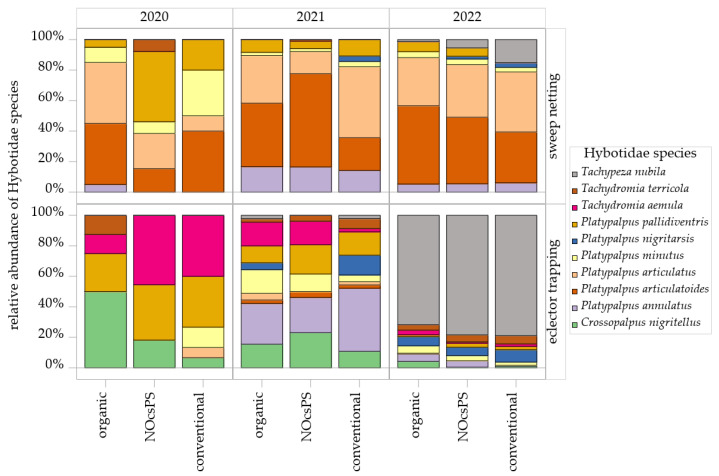
Histograms of Hybotidae species as determined in sweep netting and eclector trapping within management systems—organic, NocsPS, and conventional—for harvest years 2019/2020–2021/2022. Data shown are the top 10 species (≥5 individuals/year). Species are depicted by blocks in different colors.

A total of 25 species belonging to the family Hybotidae were recorded over the three experimental years. Upon closer examination of the species composition across the management systems, it became evident that significant shifts occurred throughout the sampling period, with notable differences in species diversity across the various collection methods (Table S3). In the season of 2020, sweep netting consistently captured the same dominant species across all management systems: *Platypalpus articulatoides* (Frey, 1918), *Platypalpus articulatus* (Marquart, 1827), *Platypalpus pallidiventris* (Meigen, 1822), and *Platypalpus minutus* (Meigen, 1804). In particular, *P. articulatoides* and *P. articulatus* contributed a high proportion to the total collections in the organic management system, with *P. articulatus* also showing high proportions in the NOcsPS management system and *P. articulatoides* in the conventional management system (Figure 5). In eclector trapping, *Tachydromia aemula* (Loew, 1864) emerged as the most dominant species in both NOcsPS and conventional management systems. Species such as *Crossopalpus nigritellus* (Zetterstedt, 1842), *Platypalpus longiseta* (Zetterstedt, 1842), and the diminutive *Stilpon nubila* (Collin, 1926) were exclusively detected via eclector trapping and were absent in sweep netting (Table S3). Notably, *P. pallidiventris* was consistently a dominant species across all eclector samples of all management systems (Figure 5).

In the season of 2021, the dominance of *P. articulatoides* and *P. articulatus* was confirmed across all management systems by sweep netting. *Tachypeza nubila* (Meigen, 1804) was recorded for the first time in this study and, notably, became the most dominant species in the following year, yielding the highest individual counts in eclector trapping (Figure 5). *Platypalpus annulatus* (Fallen, 1815) attained substantial population levels, particularly within the NOcsPS management system (Figure 5). Within the eclector trapping, *P. annulatus* emerged as the most dominant species. *P. pallidiventris* and *C. nigritellus* were also dominant species obtained by this sampling technique.

Throughout 2022, *P. articulatus* and *P. articulatoides* continued to be the most dominant species in sweep netting (Figure 5). During this year, the relative abundance of *T. nubila* increased, particularly in the NOcsPS management system, whereas the abundance of *P. pallidiventris* decreased. In eclector trapping, *Platypalpus nigritarsis* (Fallen, 1816) was the second most common species after *T. nubila*, ranking as subdominant across all management systems. *Platypalpus excisus* (Becker, 1907) and *Platypalpus brachystylus* (Bezzi, 1892) were recorded for the first time in this study by eclector trapping, appearing only in NOcsPS and conventional management systems (Table S3).

### 3.3. Predator–Prey Relationships

To assess predator–prey correlations, it is essential to consider the composition and relative abundance of potential prey taxa captured by both collection methods. The prey community consisted of five families within the Brachycera, including Agromyzidae and Chloropidae, and Nematocera, including Cecidomyiidae, Sciaridae, and a group of other small-bodied Nematocera (<7 mm) (Table 4).

To determine whether a correlation existed between the presence of prey and the occurrence of Hybotidae in the management systems, a Spearman correlation analysis using Bonett and Wright transformation for calculation of the confidence intervals was conducted for both collection methods.

Sweep netting revealed positive predator–prey associations in all management systems: NOcsPS (r (100) = 0.32629, CI [0.13363, 0.49518], *p* = 0.00115) organic (r (100) = 0.34601, CI [ 0.15478, 0.51225], *p* = 0.00056) and conventional (r (100) = 0.31341, CI [0.11991, 0.48397], *p* = 0.00182).

The higher the value for the number of individuals of prey was, the higher the number of Hybotidae and vice versa. No significant correlations were detected in eclector trapping data for any management system: NOcsPS (r (48) = –0.0001, CI [–0.2842, 0.2841], *p* = 0.9996), organic (r (47) = 0.1949, CI [–0.1006, 0.4587], *p* = 0.1946), and conventional (r (47) = 0.0073, CI [–0.2805, 0.2939], *p* = 0.9613).

### 3.4. Measurement of Soil Moisture

**Figure 6:**
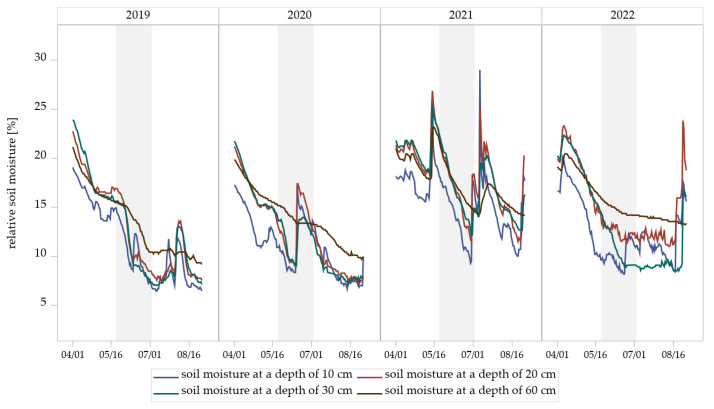
Relative soil moisture dynamics at four depths (10, 20, 30, and 60 cm) measured by soil sensors in the experimental field Dahnsdorf (2019–2022). The gray areas highlight the measured soil moisture during the sampling period of Hybotidae.

Measurements from soil sensors at the experimental field revealed seasonal dynamics of relative soil moisture at all four depths (10, 20, 30, and 60 cm) (Figure 6). Soil moisture generally declined from early April to mid-August in all years of trial, followed by short-term increases associated with rainfall events (Table S4). The extent and timing of these fluctuations varied between years and soil depths. Shallow layers (10 cm and 20 cm) exhibited more pronounced and rapid declines in soil moisture compared to deeper layers (30 cm and 60 cm), which responded more gradually to surface drying and wetting.

## 4. Discussion

The objective of this study was to gain insights into predatory Hybotidae communities and the ecological impact of pesticide application on Hybotidae–prey dynamics within winter wheat management systems. It is anticipated that the findings will contribute to the ongoing debate on sustainable agricultural practices and natural pest control by promoting biodiversity and ecological stability.

In total, 1159 Hybotidae individuals were collected during the study, with 417 collected by sweep netting and 742 by eclector trapping. Of this large number, only six individuals could not be reliably identified to species level due to the absence of distinct morphological characteristics. Among the six hybotids subjected to genetic identification by COI barcoding, three did not have sufficiently high sequence identities with BOLD or GenBank datasets to reliably identify them to the species level despite having high-quality sequence results. For this situation, two reasons may apply: (1) the species to be identified is unknown or has not yet been described, or (2) the species to be identified is known, but nobody has yet attempted to produce COI sequences and submit them to the databases. In either case, such specimens could not be included in the analysis.

The results did not reveal consistent management system effects on the number of collected Hybotidae in both collection methods. Although differences among management systems were observed, these were not statistically significant, indicating that Hybotidae abundance was largely unaffected by pesticide avoidance or fertilization regime within the three-year period. The newly implemented hybrid NOcsPS system, which combines the absence of chemical synthetic pesticides with an optimized nitrogen fertilization strategy, showed a slightly higher number of captured Hybotidae individuals in sweep netting compared to the organic, but a substantially higher abundance compared with the conventional management system. The observed trend suggests a potentially positive, though not statistically verified, influence of reduced pesticide use and optimized fertilization strategy on Hybotidae activity. In agreement with findings from the same project published elsewhere, the order Diptera overall appeared to benefit most from the NOcsPS management system [40]. The reduced disturbance in the extensive management systems may have minimized both direct toxic effects and indirect impacts via food web alterations, supporting prey populations and habitat structure, thereby promoting Hybotidae occurrence [56,57].

Unexpected patterns emerged in the conventional management system results: despite the use of chemical synthetic pesticides, this management system showed high abundances of Hybotidae in the eclector trapping, surpassing the numbers of collected individuals in the organic, but not in the NOcsPS management system. This finding was inconsistent with the results of other studies, which indicated a reduction in the abundance of Hybotidae and other species with increased application of pesticides and fertilizers [58,59,60]. However, it is noteworthy that some other insect taxa appear to benefit from conventional farming systems [61]. The number of individuals in the conventional management system, collected by sweep netting, was 46% lower than in the organic and 53% lower than in NOcsPS management systems, suggesting that some individuals may have migrated into neighboring plots.

The discrepancies in outcomes between the sweep netting and eclector trapping highlight the importance of employing a combination of collection methods to obtain a comprehensive representation of insect abundance and diversity and therefore a more detailed overview of the species community [32,62]. Since sweep netting is best suited for the rapid collection of flying and easily dislodged insects, it is ideal for open fields and is known to be suitable for comparing abundance and species richness of small arthropods, as Hybotidae, occurring in similar vegetation types [63]. Eclector trapping is more effective for capturing ground-dwelling insects that develop in and emerge from soil, and are frequently used in studies on Empidoidea [34].

Differences in the number of individuals collected by sweep netting and eclector trapping under conventional management may, at least in part, result from direct and indirect population disturbances induced by pesticide application, such as the disruption of predator–prey interactions and associated trophic dynamics [64,65]. The level of insecticide application can impact the presence of prey of Hybotidae, as demonstrated for other families of insects [66]. The conventional management system was conducted in accordance with good agricultural practices, with insecticides only applied when pest thresholds were exceeded. Consequently, no insecticides were employed for the management of Chloropidae and Agromyzidae throughout the study period. Broad adoption of this threshold-based application strategy could positively affect bioindicators such as Hybotidae, since insecticides targeting Chloropidae, Agromyzidae, and Cecidomyiidae usually consist of pyrethroids, which are known to negatively affect non-target organisms, including Hybotidae [67,68]. Insecticide applications occurred only in harvest year 2021/2022 (autumn and early spring) to lower ground beetle populations, while no such treatments were conducted in the previous two years. The insecticide employed, Karate Zeon (active ingredient: lambda-cyhalothrin), is a contact and ingestion insecticide. It can have a negative influence on Diptera, not only by increasing mortality but also through sublethal effects, including impaired behavior and reduced oviposition [69,70,71]. Lambda-cyhalothrin is stable in sunlight and has a long-lasting effect on plant surfaces. However, it is unlikely to affect Sciaridae, as the adults do not feed on plant tissues and remain near the soil surface. Cecidomyiidae feed on plant tissues but typically appear in late April, when the insecticide’s effect has already declined [72]. Larvae of the families Chloropidae and Agromyzidae overwinter in plant sheaths and emerge in late May or early June, thereby avoiding exposure to the insecticides in early spring. Moreover, the efficacy of many insecticides against Agromyzidae is limited, as the eggs and larvae are protected within plant tissues [73]. It is crucial to consider that insecticide application often impacts natural antagonists of Agromyzidae (i.e., parasitoid wasps) more severely than the pest itself, thereby disrupting natural control dynamics. Such imbalances can lead to secondary outbreaks, where suppression of antagonists allows Agromyzidae populations to reach damaging levels, even when not directly targeted by insecticide programs [74,75,76]. The life cycles of these Diptera provide an explanation for the limited direct impact of the insecticide on their populations in this study.

Herbicides can exert lethal and sublethal effects across a broad range of insect taxa, primarily through indirect pathways such as altered host plant availability or weed removal [77]. Direct effects of herbicides on Hybotidae and their prey are currently undocumented. Nevertheless, these processes showed an influence on certain Diptera populations, such as Tachinidae, by modifying habitat structure, plant traits, or through synergistic interactions with insecticides [78,79,80].

While these interactions remain poorly understood, they may nonetheless contribute to subtle differences in community structure and diversity among management systems, particularly within Hybotidae assemblages.

Considering the results of alpha biodiversity indices, including the Shannon index, evenness, and Berger–Parker index, the diversity of Hybotidae species did not appear to be directly influenced by the application of pesticides or fertilization regimes. No statistically significant differences were detected among management systems across any of the alpha diversity indices. Regarding sweep netting, the NOcsPS management system tended to show slightly higher Shannon diversity but lower evenness and dominance values compared to the other treatments. The organic system displayed the opposite trend, with lower Shannon diversity but higher evenness and dominance indices, while the conventional system consistently occupied an intermediate position. Eclector trapping revealed a similar pattern, with only minor variations in diversity and dominance among the three management systems. Although none of the tendencies were statistically significant, they may point toward subtle shifts in community structure under different management regimes and highlight the need for further research to better understand the underlying ecological dynamics. The observed variability in diversity indices suggests the presence of complex interactions within the agroecosystem, which are influenced by a multitude of factors beyond the direct application of pesticides and fertilization practices. The Shannon index is one of the most widely used and best-known measures of species diversity [81]. Together with evenness, it provides a generally reliable assessment of α-diversity in agricultural systems, including wheat fields, provided that sampling methods are standardized and sample sizes are comparable. However, results can be strongly influenced by sampling techniques and effort, as different methods (e.g., sweep netting vs. eclector trapping) target distinct functional groups and activity strata. Both indices describe only taxonomic richness and evenness, without accounting for functional or trophic diversity, which is particularly relevant in simplified agroecosystems. It has also been argued that the understanding of diversity lies not in the numerical form of indices, but in the ecological meaning of the variation in abundance values that underlie their calculation [82]. Temporal and spatial variability in wheat fields can further obscure diversity patterns, complicating interpretation.

Notably, the harvest year 2021/2022 was characterized by a markedly higher collection rate across all management systems, likely improving the robustness and reliability of the biodiversity assessments. This increased sampling success or population density may have allowed for more accurate detection of management-related effects and community patterns, highlighting the importance of sufficient sample sizes in ecological monitoring. Overall, these findings underscore that interpreting diversity indices should be interpreted cautiously, within the framework of sampling design and ecological context [83].

In total, 25 species were collected during the study period, with 14 species captured by sweep netting and 21 species by eclector trapping. Consequently, 11 species were detected exclusively in the eclector samples, while four species were found only in the sweep netting samples, again highlighting the necessity of integrating several collection techniques. There are notable annual variations in the community structure in the management systems. This variability highlights the complex nature of ecological interactions and the impact of changing climatic and environmental factors on insect populations. For instance, the occurrence of *P. annulatus* is unusual and may be linked to the soil type. A pronounced effect of soil type (podzol vs. parabrown earth) on species composition and abundance–particularly in *Platypalpus* spp.–was observed in photoeclector samples collected from various localities in the German federal state of Schleswig–Holstein [84]. In that study, *P. minutus* showed abundances of 4.3 individuals per square meter and month in cereal fields in organically managed plots on parabrown clay. As opposed to that species, *P. pallidiventris* and *P. longiseta* are commonly found in the fauna of wheat fields, as are the species *P. nigritarsis* and *P. excisus*. The emergence of the two *Euthyneura* species, *Euthyneura myrtilli* and *Euthyneura inermis*, was likely to be incidental, since they are supposed to appear primarily in response to the availability of floral resources [66].

It should be noted that our findings revealed a Hybotidae community that is characteristic of light-open habitats. It seems that the key generalist predator genus, *Drapetis*, is underrepresented in our study, particularly in comparison to the findings of other investigations [85]. *T. nubila* was almost exclusively captured in the eclector trapping and in particularly high numbers. Species of the genus *Tachypeza* are known to move continuously on the ground rather than on vegetation and to fly only rarely and over very short distances. *T. terricola* and *T. aemula* were likewise recorded exclusively in the eclector trapping. These species also exhibit limited flight activity and capture their prey while running on the soil surface or within the ground-level vegetation [21]. It can therefore be assumed that these species do not leave or actively immigrate into the plots, but remain within their immediate habitat.

It was expected that untreated plots of organic and NOcsPS management systems would exhibit higher species diversities and abundances of individuals than pesticide-treated plots [86]. The general assumption that conventional agriculture with pesticide application will lead to a loss of species biodiversity was not confirmed by the methodological approach practiced here. For example, the number of individuals of the species *P. articulatoides* was found to decline over the years in the conventionally managed plots. Instead, other species, such as *P. pallidiventris* and *P. minutus*, became dominant. These results may indicate that different species exhibit disparate responses to insecticide treatments. However, alternative explanations are also plausible, given the lack of knowledge about the autecology and population ecology of these species. If a *T. nubila* female deposits her eggs within a localized area and natural egg predators are temporarily suppressed by a short-term insecticide application, a higher proportion of larvae may survive and successfully develop. Even accounting for natural mortality, this could lead to an increased emergence rate, which may help explain the elevated number of *T. nubila* individuals recorded in the season of 2022.

Consistent positive associations were observed between Hybotidae as predators and Chloropidae, Agromyzidae, Sciaridae, and Cecidomyiidae as prey in sweep netting of all management systems. These relationships suggest that Hybotidae populations tend to increase with greater prey availability, indicating efficient predation and the potential contribution of natural pest control processes. While such correlations do not confirm causality, they provide strong evidence of ecological linkages between Hybotidae and their prey communities. In contrast, the eclector trapping data revealed no significant predator–prey correlations. This difference can be explained ecologically: only two of the identified prey families, Sciaridae and Cecidomyiidae, overwinter and emerge from the soil, whereas Agromyzidae and Chloropidae overwinter within damaged plant tissues of wheat or on alternative winter hosts [73,87,88]. Hybotidae larvae are predaceous on other dipteran larvae [20,32]; thus, it is plausible that they feed on larvae of Sciaridae and Cecidomyiidae. Such interactions cannot be confirmed within the scope of the present study.

The demonstrated year-to-year variability in Hybotidae abundance in eclector trapping indicates that multiple environmental and ecological factors may be exerting an influence on these Diptera. The observed increase in Hybotidae abundance lacks a straightforward explanation but is likely linked to indirect factors, such as variations in microclimatic conditions, prey availability, and predator presence across management systems. Soil moisture measurement showed a seasonal decline in all years, with stronger fluctuations in the upper soil layers (10–20 cm) than in the deeper layers (30–60 cm). Extended dry periods in 2019 and 2020 contrasted with higher and more variable moisture in 2021 and 2022. Sharp moisture peaks prior to and during the sampling period may have promoted synchronized pupation after dry stress phases. While temperatures remained similar between years, reaching a peak of 19–20 °C in June and July, precipitation levels differed markedly (Table S4). The cumulative rainfall in 2021 from the beginning of May to mid-July was the highest of the study period (≈200 mm), resulting in moist conditions, favorable for both prey and predator development (Table S4). Given that Hybotidae larvae develop in humid soil or water [14,21], soil moisture in the uppermost layers likely plays a key role in regulating emergence, while short-term rewetting events may act as triggering cues. Climatic factors are known to affect insect population dynamics directly and indirectly by influencing survival, behavior, and developmental cycles [89]. Adequate moisture is essential for the survival and development of many insect species, including the prey of Hybotidae [90]. It is likely that the weather conditions in harvest year 2020/2021 created favorable conditions for insect populations [91,92], producing a cascade effect in which increased prey densities led to higher reproductive output in predators [93]. The elevated precipitation levels observed in 2021 may have resulted in soil conditions that were beneficial to the growth of fungal mycelia, thereby supporting the development of sciarid larvae and other prey species of Hybotidae [94,95]. The enhanced accessibility of prey furnished a substantial food source for adult Hybotidae, consequently facilitating the production of more eggs, which in turn led to a greater number of larvae developing and surviving through to the harvest year 2021/2022. Such dynamics are consistent with a typical lag effect in population dynamics, where favorable conditions in one year can lead to increased populations in the following season [96,97].

At the landscape level, the limited size of the experimental plots (5 × 10 m) and their proximity (10 m) to other crop plots must be acknowledged. Previous studies on Empidoidea, including Hybotidae, have shown that species respond differently to environmental heterogeneity [34]. Although neighboring crops may influence insect populations, the plots were sufficiently large to provide representative samples of the Hybotidae community associated with this wheat type. The influence of the surrounding landscape is inherent to field studies and reflects the natural environmental context in which these insects occur. In this context, nearby moist habitats such as forest edges or semi-natural grasslands may have contributed to the observed abundance of Hybotidae by providing favorable conditions or alternative breeding sites [98,99,100], from which individuals may subsequently have dispersed into the wheat stands. Considering the typical behavior of Hybotidae, characterized by slow, low flight, limited dispersal, and a sit-and-wait predatory strategy [21,34], it is reasonable to assume that individuals rarely move between plots, except when seeking habitats with higher prey availability.

Finally, differences in seed distribution between normal sowing and single-grain placement may have affected microhabitat structure. Wheat stand density, its data not available for analysis here, likely influenced light, temperature, and humidity near the soil surface. Denser stands may create more shaded, humid microclimates that serve as suitable habitats for larval development in some species and may function as resorts from which adults colonize more exposed areas [101].

The comparative analysis of the Hybotidae community across different management systems and years collectively demonstrates the substantial influence of agricultural and ecological factors on insect populations. Naturally, the system under study is highly complex, and the interpretation of results remains challenging. The multifactorial nature of such experiments can obscure long-term ecological responses that may only become evident in subsequent years, extending beyond the typical three-year observation period.

## 5. Conclusions

The findings of our study demonstrate that Hybotidae communities in winter wheat are shaped by a complex interplay of agricultural management and environmental factors rather than by pesticide regimes alone. Although no statistically significant differences were detected among management systems, subtle trends suggest that organic and NOcsPS systems, which restrict the use of synthetic chemical pesticides, may support more balanced and stable predator–prey dynamics. The results highlight that short-term studies may not capture the full ecological responses of Hybotidae, and long-term, multi-site research is required to verify potential management-related effects. The agricultural landscape provides highly specific conditions that certain species can exploit optimally or within which they can tolerate substantial disturbances. However, our understanding of the underlying ecological drivers remains very limited. While this study offers valuable insights into sustainable crop management at the field scale, the broader success of alternative systems such as NOcsPS depends largely on their consistent implementation at the farm and landscape levels. Future research should combine long-term population monitoring with detailed climatic data and direct observations of predator–prey relationships to enhance our understanding of Hybotidae ecology. Moreover, the potential effects of crop management factors, including crop rotation, spatial plant distribution, and fertilization strategies, should be evaluated to optimize agricultural productivity while maintaining ecological integrity.

## Figures and Tables

**Table 1:** Hybotidae abundance comparison between management systems using a generalized linear model with a negative binomial distribution and the SIMULATE method for multiple testing (α = 0.05). The F, df, and *p*-values are provided for the fixed effect ‘management system’.

	Sweep Netting		Eclector Trapping	
fixed effect	F(df1,df2)	*p* value	F(df1,df2)	*p* value
management system	F(2,128) = 1.73	0.1808	F(2,73.01) = 0.44	0.6460
SIMULATE method				
for Multiple Comparisons				
NOcsPS vs. conventional		0.1629		0.8709
NOcsPS vs. organic		0.5639		0.8977
conventional vs. organic		0.5990		0.6182

**Table 2:** Total number of Hybotidae individuals collected in sweep netting and eclector trapping across three management systems and years (2019/2020–2021/2022).

Collection Method	Management System	Harvest Year	Total
Sweep netting	organic	2019/2020	23
		2020/2021	53
		2021/2022	79
	NOcsPS	2019/2020	16
		2020/2021	105
		2021/2022	57
	conventional	2019/2020	11
		2020/2021	36
		2021/2022	37
Eclector trapping	organic	2019/2020	10
		2020/2021	48
		2021/2022	166
	NOcsPS	2019/2020	12
		2020/2021	29
		2021/2022	242
	conventional	2019/2020	16
		2020/2021	47
		2021/2022	197

**Table 3:** Alpha diversity measurements of Hybotidae. Comparisons were performed between management systems using a generalized linear model with a negative binomial distribution and the SIMULATE method for multiple testing (α = 0.05). The F, df, and *p*-values are provided for the fixed effect ‘management system’. Hs = Shannon index; E = evenness; d = Berger–Parker index.

		Sweep Netting		Eclector Trapping	
Alpha diversity measures	Fixed effect	F(df1,df2)	*p* value	F(df1,df2)	*p* value
Hs	management system	F(2,128) = 0.04	0.9651	F(2,100) = 0.02	0.9780
	NOcsPS vs. conventional		0.9919		0.9821
	NOcsPS vs. organic		0.9647		0.9831
	conventional vs. organic		0.9939		0.9999
E	management system	F(2,51.43) = 0.45	0.6396	F(2,54.46) = 0.09	0.9123
	NOcsPS vs. conventional		0.9478		0.9696
	NOcsPS vs. organic		0.6277		0.9797
	conventional vs. organic		0.8585		0.9031
d	management system	F(2,117.4) = 0.14	0.8666	F(2,85.45) = 0.2	0.8219
	NOcsPS vs. conventional		0.8707		0.8145
	NOcsPS vs. organic		0.9130		0.8991
	conventional vs. organic		0.9877		0.9853

**Table 4:** Abundance of hybotid prey in eclector trapping and sweep netting (2019/2020–2021/2022).

	Management System	Agromyzidae	Chloropidae	Cecidomyiidae	Sciaridae	Other Nematocera (<7 mm Body Size)
Sweep netting	organic	56	869	45	377	121
	NOcsPS	56	1847	36	397	39
	conventional	57	1766	83	434	39
Eclector trapping	organic	19	1400	717	618	6
	NOcsPS	33	2199	584	1001	17
	conventional	30	2153	565	1189	5

## Data Availability

The raw data supporting the conclusions of this article will be made available by the author upon request.

## Supplementary Materials

The following supporting information can be downloaded at: https://www.mdpi.com/article/10.3390/insects16121263/s1, Table S1. Field book 2020–2022; Table S2. Drilling scheme of the wheat plots; Table S3. Summary of Hybotidae species distribution across sampling methods and management systems during the study period; Table S4. Monthly mean temperature values and monthly precipitation, Dahnsdorf 2020–2022.
